# Sorafenib-Based Drug Delivery Systems: Applications and Perspectives

**DOI:** 10.3390/polym15122638

**Published:** 2023-06-09

**Authors:** Lingyun Wang, Meihuan Chen, Xueguang Ran, Hao Tang, Derong Cao

**Affiliations:** 1 Key Laboratory of Functional Molecular Engineering of Guangdong Province, School of Chemistry and Chemical Engineering, South China University of Technology, 381 Wushan Road, Guangzhou 510641, China; jasmine80420@163.com (M.C.); haotang@scut.edu.cn (H.T.); drcao@scut.edu.cn (D.C.); 2 Institute of Animal Science, Guangdong Academy of Agricultural Sciences, Ministry of Agriculture Key Laboratory of Animal Nutrition and Feed Science in South China, State Key Laboratory of Livestock and Poultry Breeding, Guangzhou 510641, China; rxg59@aliyun.com

**Keywords:** sorafenib, multi-kinase inhibitor, nanodelivery systems, hepatocellular carcinoma (HCC), nano-sized carriers, tumor treatment

## Abstract

As a Food and Drug Administration (FDA)-approved molecular-targeted chemotherapeutic drug, sorafenib (SF) can inhibit angiogenesis and tumor cell proliferation, leading to improved patient overall survival of hepatocellular carcinoma (HCC). In addition, SF is an oral multikinase inhibitor as a single-agent therapy in renal cell carcinoma. However, the poor aqueous solubility, low bioavailability, unfavorable pharmacokinetic properties and undesirable side effects (anorexia, gastrointestinal bleeding, and severe skin toxicity, etc.) seriously limit its clinical application. To overcome these drawbacks, the entrapment of SF into nanocarriers by nanoformulations is an effective strategy, which delivers SF in a target tumor with decreased adverse effects and improved treatment efficacy. In this review, significant advances and design strategies of SF nanodelivery systems from 2012 to 2023 are summarized. The review is organized by type of carriers including natural biomacromolecule (lipid, chitosan, cyclodextrin, etc.); synthetic polymer (poly(lactic-*co*-glycolic acid), polyethyleneimine, brush copolymer, etc.); mesoporous silica; gold nanoparticles; and others. Co-delivery of SF and other active agents (glypican-3, hyaluronic acid, apolipoprotein peptide, folate, and superparamagnetic iron oxide nanoparticles) for targeted SF nanosystems and synergistic drug combinations are also highlighted. All these studies showed promising results for targeted treatment of HCC and other cancers by SF-based nanomedicines. The outlook, challenges and future opportunities for the development of SF-based drug delivery are presented.

## 1. Introduction

Sorafenib (SF) is a US Food and Drug Administration (FDA)-approved molecular-targeted chemotherapeutic agent [1,2,3,4], which is used as a clinic standard drug for hepatocellular carcinoma (HCC). The overall patient survival is increased by delaying the pathologic progression. SF is also an oral multikinase inhibitor by inhibiting the proliferation of tumor cells, neoplastic angiopoiesis, angiogenesis and invasion of cancer cells. So, SF is regarded as an effective chemotherapeutic agent against various types of tumors [5]. However, due to the high dose, poor solubility (25 ng/mL for SF free base in water), low bioavailability, dose-limiting side effects (diarrhea, anorexia, heart attack and infection, etc.) and possible drug resistance, the therapeutic application of SF is greatly restricted [6]. At the same time, pH-dependent SF is more soluble in an acidic environment because of the presence of amide and pyridine moieties. The aqueous solubility of commercial SF tosylate salt (Nexavar^®^ by Bayer Pharmaceuticals, Leverkusen, Germany) is improved (65 ng/mL), but it is still poorly soluble in water [7]. Sorafenib tosylate has been authorized for renal cancer, hepatocellular cancer and differentiated thyroid cancer. The suggested dose in oral usage is 400 mg, which is equivalent to two pills each day (200 mg/tablet) [8]. However, some shortcomings of sorafenib tosylate, such as low drug solubility, low drug permeability, cytotoxicity, side effects, and drug resistance across cancer cells, are still present.

**Figure 1:**
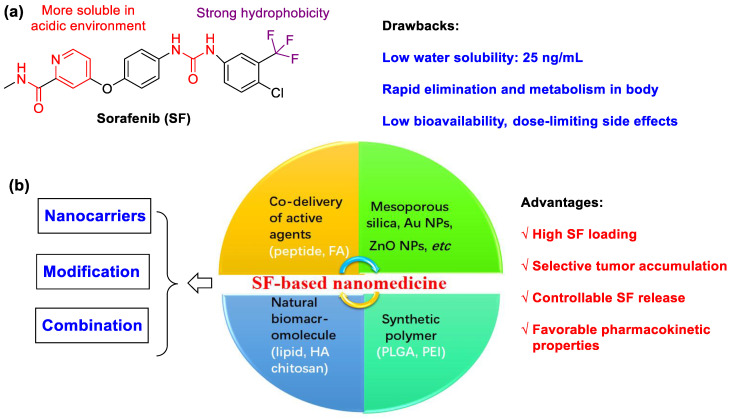
(**a**) The chemical structure of SF. (**b**) The design strategies of SF-based nanomedicine. Nanocarriers to improve release efficiency and bioavailability and actively target tumor tissues. Modification methods to increase the targeting and responsiveness of SF-based NPs. Combination therapies to Enhance treatment efficacy.

Nanomedicine, via the delivery of drugs to tumor cells in a targeted fashion, is a promising strategy to solve these drawbacks [9,10]. Until now, the use of amphiphilic block copolymers as nanocarriers to generate colloidal delivery systems for hydrophobic drugs has been widely investigated. It is important to control particle size and distribution, morphology, zeta-potential, drug loading, encapsulation efficiency and process yield, which significantly affect drug pharmacokinetic properties, treatment outcome, biosafety, etc. There are distinct advantages such as: (1) carrying large amounts of drugs; (2) having prolonged circulation time; (3) facilitating selective tumor accumulation via the enhanced permeability and retention (EPR) effect; and (4) controlling drug release to target tissues. To achieve this aim, the entrapment of SF into nano-sized carriers affords SF-based nanomedicine, which is beneficial to reduce its side effects [11,12,13,14,15,16]. In this review, we focus on the recent advances of the nanocarrier-based delivery of SF (liposome, nanoparticle, micelle, emulsion, etc.) including preparation, mechanism of drug release, treatment efficiency and so on. The review is organized by type of carriers, including natural biomacromolecule (lipid, chitosan, cyclodextrin), synthetic polymer (poly(lactic-*co*-glycolic acid), polyethyleneimine, brush copolymer), mesoporous silica and others. Co-delivery of SF and other active agents (glypican-3, hyaluronic acid, apolipoprotein peptide, folate, superparamagnetic iron oxide nanoparticles) for targeted SF nanodrugs as well as other chemotherapeutic drugs are also highlighted (Figure 1), which show great potential to fight against various types of cancers. This review presents the latest advances, outlook, challenges and future opportunities in SF-loaded NPs to improve cancer treatment. We hope this review provides an insight and prospects in the design and preparation of ideal SF nanoformulations for clinical cancer therapy.

### SF Inhibition Mechanism in HCC and Other Cancers

Three underlying mechanisms have been found to support SF inhibition mechanism in HCC [17]. First, SF blocks HCC cell proliferation by inhibiting BRaf and Raf1/c-Raf serine/threonine kinase phosphorylation in the mitogen-activated protein kinase pathway. Second, SF induces apoptosis by reducing elF4E phosphorylation and downregulating Mcl-1 levels in tumor cells. Third, SF prevents tumor-associated angiogenesis by inactivating vascular endothelial growth factor receptors (VEGFR-2 and -3) and the platelet-derived growth factor receptor-β. In addition, SF is also shown to induce apoptosis through downregulation of Mcl-1 in many cancer types. Hence, SF as a single agent has shown promising activity in some cancers such as renal cell carcinoma (RCC) and thyroid cancers. Clinical trials have demonstrated the effectiveness and relative safety of SF, and thus, the drug is used in unresectable HCC. However, resistance to SF and serious side effect is shown by many patients. Many studies have combined SF with other treatments in an effort to increase its effects, reduce the necessary dose or overcome resistance.

## 2. SF-Based Nanomedicine

### 2.1. Biomacromolecule as Nanocarrier

As a kind of potential candidates for encapsulating hydrophobic drugs, nanostructured lipid carriers (NLCs) possess suitable size, good oral absorption, high biocompatibility, low toxicity and immunogenicity [18,19,20,21]. For example, the commercially pegylated liposomal doxorubicin (Doxil/Caelyx) and liposomal daunorubicin (Daunoxome, Galen) have been FDA approved as antitumor formulations. NLCs can be classified into solid lipid nanoparticles (SLNs) and liquid lipid nanoparticles (LLNs). The major difference between SLNs and LLNs is their lipid components. SLNs are prepared from solid lipids, whereas NLCs are modified by adding liquid lipids [20]. Both SLNs and LLNs are considered to be safe carriers since they are produced from physiological and biodegradable lipids. They can increase the stabilities of active ingredients and drug. However, LLNs exhibit higher drug loading capacities than SLNs, and lipid crystallization is avoided during drug storage because of the presence of liquid lipids, thus preventing drug expulsion. Until now, liposomes have been widely used as suitable nanovectors for the SF delivery since SF has high hydrophobicity and good lipid affinity.

Bondìa and coworkers found that an NLC mixture (from solid and liquid lipids) would be a better nanocarrier than solid lipids alone, where higher drug loading capacity and longer-term stability were shown [22]. In vitro results indicated that more efficiently inhibited HCC cell growth than free SF (70% vs. 40% at 10 mM, and 85% vs. 55% at 20 mM) were obtained, leading to enhanced anti-tumor activity. When SF-loaded SLNs were prepared by combined high-speed shearing with ultrasonic treatment, high average encapsulation efficiency (EE) and drug loading (DL) of 89.87 and 5.39%, respectively, were shown [23]. Due to good liver-targeting capability, a 2.20-times higher average drug selectivity index value was found compared to that of the free SF-suspension. After oral administration, SF is metabolized in the mucosa of the small intestine and in the liver, where it is transformed into eight metabolites. When SF was encapsulated in SLNs matrix, SF was protected from metabolism or slowed down SF release from SLNs. Thus, SF remained in systemic circulation for a prolonged duration. Therefore, reducing the dosage of SF-SLNs could be considered to achieve the same pharmacological effects as the SF-suspension. As a result, a low side effect via oral administration was obtained by the protective lipid matrix and improved bioavailability.

The charged nucleolipids possess good stability, non-toxicity and self-assembly properties. Benizri et al. reported charged SF-loaded SLNs entrapped by nucleolipids with improved water solubility of SF (>120 μM) and parallelepiped microstructure with 200 nm size [24]. Due to improved SF solubility, enhanced anticancer activities on four different cell lines (liver and breast cancers) were shown compared to free SF. Meanwhile, Ahiwale et al. used nanocarriers compressing 1,2-dioleoyl-sn-glycero-3-phospho-L-serine sodium salt and cholesterol for oral administration of SF tosylate [25]. The entrapment efficiency, particle size and zeta potential of 85.55%, 470 nm and −44.5 mV, respectively, was shown. The 2.18-fold improvement in oral bioavailability and a pronounced effect on liver cancer was found. In one case, SF was incorporated into the hydrophobic lipidic bilayer with a encapsulation efficiency rate of 75.35% [26]. In vitro cytotoxicity effect revealed the IC_50_ values decreased by ~30% at 24 h (12.25 µM for SK-HEP-1 and 11.61 µM for HepG2).

Ye et al. reported distearyl phosphatidylethanolamine polyethylenelycol (DSPE-PEG) modified nanoliposomes for SF delivery, followed by chemically coupled anti-VEGFR antibody as a targeting moiety [27]. The SF entrapment, loading efficiency and binding efficiency of the antibody were 92.5%, 18.5% and 23.1%, respectively. When resulting SF-based nanoliposomes were intravenously injected into the mice, the half time was nearly 10 h, indicative of long circulation capability. Better tumor inhibitory effect was observed than that of free SF due to the combination of long-circulating nanoliposomes, targeting antibodies and improved therapeutic efficiency. Liu et al. investigated the interaction of nanodrug platinum nanoparticle–SF with VEGFR2 [28].

**Figure 2:**
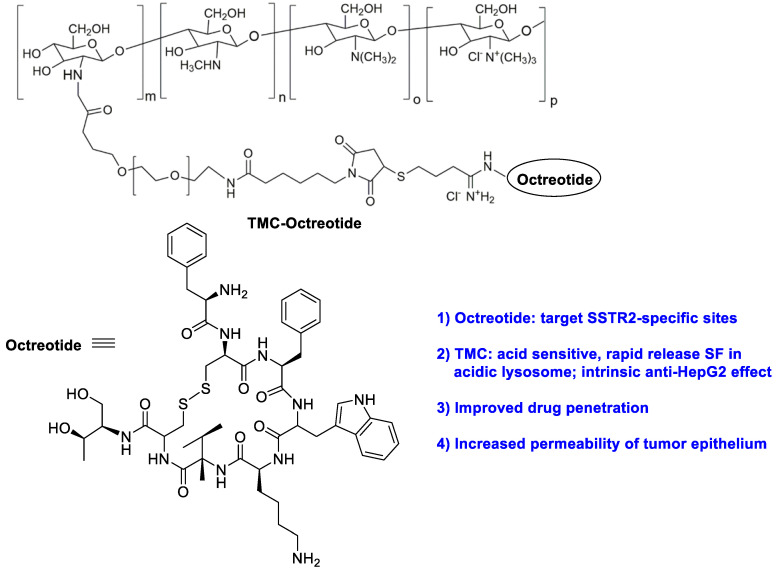
The chemical structure of trimethyl chitosan (TMC) -octreotide.

As a type of natural polymer, chitosan has good biodegradability, non-toxicity, and good affinity for adsorbing drugs. For instance, chitosan/heparin-immobilized Pluronic NPs were formulated to deliver SF, leading to enhanced antitumor efficacy of SF in gastric cancers [29]. Varshosaz et al. developed PEGylated trimethyl chitosan (TMC) emulsomes (EMs) conjugated with octreotide (TMC-octreotide) for targeted delivery of SF by the emulsion evaporation method (Figure 2) [30]. The optimized SF-loaded EMs had the particle size of 127 nm, zeta potential of −5.41 mV, loading efficiency of 95% and drug release efficiency of 62% within 52 h. Compared to free SF and non-targeted EMs, SF-loaded targeted EMs exhibited more cytotoxicity and more accumulation in HepG2 cells. Further study indicated chemically active free amine groups in TMC had a positive effect on enhancing the stability of EMs, cell adhesion capability and retaining the targeting at the tumor area. On the other hand, due to the acidic pH character of the tumor microenvironment, pH-sensitive carboxymethyl chitosan-modified liposomes for SF and siRNA co-delivery were reported [31]. When 4.5 mg/kg of nanoformulation was administrated intravenously, the controlled SF delivery led to a reduced tumor volume.

**Figure 3:**
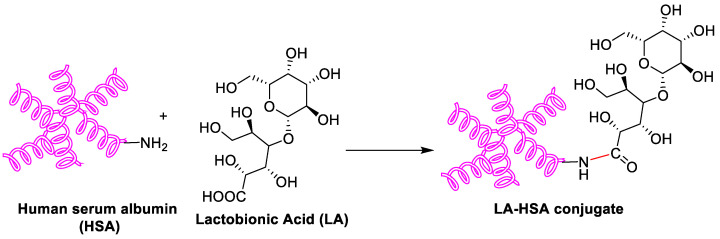
Schematic synthesis of LA-HSA conjugate.

As a biodegradable, non-toxic and non-immunogenic protein, human serum albumin (HSA) is a promising carrier with multiple cellular receptors. Lactobionic acid (LA), the ligands for asialoglycoprotein (ASGP) receptors in HepG2 cells, was conjugated to HSA by amide bond formation between LA and HSA (Figure 3) [32]. The resulting LA-HSA conjugates were used as a targeted delivery system taking SF to HepG2 cells, which showed more cytotoxicity and cellular uptake than the untargeted ones with an IC_50_ value of 1.83 and 19.07 μM, respectively. The results indicated that LA as a targeting agent had a positive effect on tumor cell killing. Similarly, Gopakumar and coworkers showed core–shell protein nanoparticle as an albumin nanocarrier for SF showed a nearly two-fold enhancement in the oral bioavailability and enhanced therapeutic efficacy [33].

**Figure 4:**
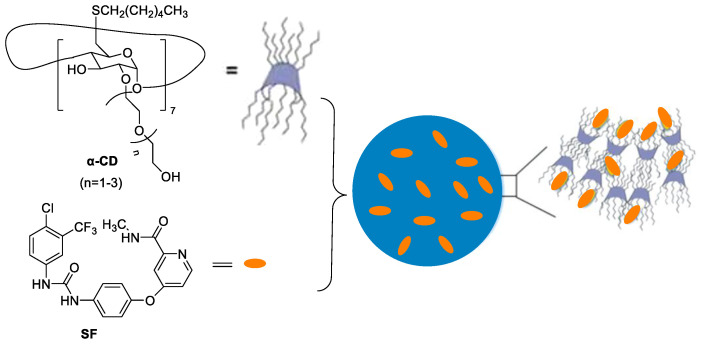
The supramolecular nanoassemblies based on α-CD and SF in aqueous solution.

Nonionic α-cyclodextrins (α-CD) are ideal carriers to deliver drugs [34]. Mazzaglia et al. reported supramolecular nanoassemblies based on α-CD and SF in aqueous solution for ablation of human HCC cells (Figure 4) [35]. The host–guest cooperation interaction between α-CD and SF produced highly water-dispersible colloidal nanoassemblies. The size of ~200 nm, negative zeta-potential (ζ = −11 mV), maximum loading capacity (~17%) and entrapment efficiency (~100%) were found. There was a slower release of SF from nanoassemblies with respect to the free SF. In addition, nanoassemblies showed low hemolytic activity and a high efficiency to inhibit the growth of three different HCC cell lines.

As a natural anionic polysaccharide, hyaluronic acid (HA) directly binds to CD44, and RHAMM receptors overexpressed on various tumor cell surfaces. Mo et al. reported PEGylated HA-coated liposome for enhanced SF delivery and anti-tumor outcome via active tumor cell targeting and prolonged systemic exposure [36]. For MDA-MB-231 cells over expressing CD44, greater cellular uptake and cytotoxicity were found. However, there were no significant differences in MCF-7 cells with low CD44 expression. Compared with SF solution, PEG-HA-SF-Lip increased the systemic exposure and plasma half-life in rats by 3-fold and 2-fold, respectively. The effective tumor growth inhibition through CD44 targeting in the MDA-MB-231 tumor xenograft mouse model was demonstrated.

SF and cisplatin (*cis*-Pt) are two chemotherapeutic drugs with different antitumor mechanisms. Zhang et al. developed pH-sensitive hyaluronic acid NPs encapsulating with SF and *cis*-Pt, where interactions between cisplatin and HA was involved [37]. The pH-sensitive property guaranteed the effective release of loaded drugs (cis-Pt and SF) in tumor sites. The HA@*cis*-Pt@SF showed prolonged circulation time and high accumulation rate, leading to exerted synergistic tumor-killing effects.

### 2.2. Synthetic Polymer as Nanocarriers

**Figure 5:**
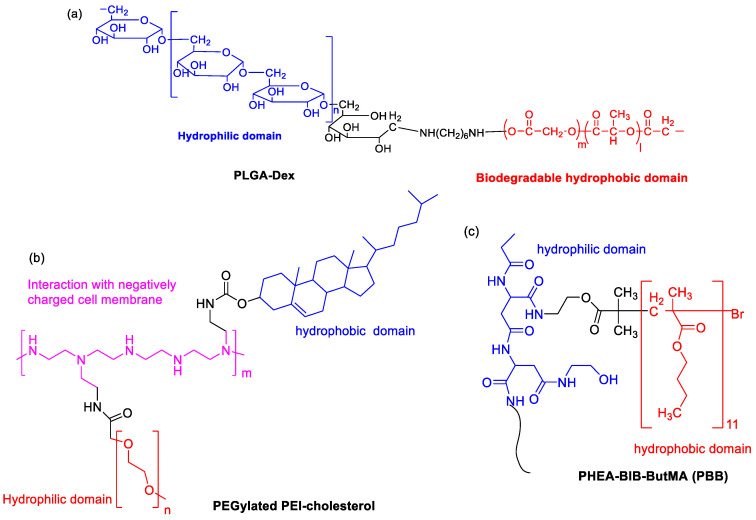
The chemical structures of (**a**) PLGA-Dex, (**b**) PEGylated PEI-cholesterol and (**c**) PBB.

Poly(lactic-*co*-glycolic acid) (PLGA) is approved by the FDA as a drug carrier due to its hydrophobic properties and biocompatibility. Kim et al. prepared hydrophilic dextran-conjugated PLGA (PLGA-Dex, Figure 5a) [38]. In this case, dextran and PLGA acted as a hydrophilic and biodegradable hydrophobic domain, respectively. The SF-incorporated PLGA-Dex NPs were fabricated via the nanoprecipitation–dialysis method. The loading efficiency was higher than 35%. An initial burst of release and a sustained release of SF was observed. A dose-dependent antiproliferative effect against HuCC-T1 tumor cells was shown. In 2016, to regulate size polydispersity and the stability of NPs in the blood circulation, a mixture of amphiphilic copolymer of poly(ethylene glycol)-*b*-PLAG and PLGA (5/5) was co-formulated to entrapped SF [39]. The blood circulation of the cargo was significantly prolonged, resulting in increased uptake by liver fibrosis in CCl_4_-induced fibrosis models. In particular, vessel normalization in the fibrotic livers was observed.

Due to the presence of primary, secondary and tertiary amines in the polymer structure, cationic polyethyleneimine (PEI) exhibits “proton sponge” effect by interaction with negatively charged cell membrane. PEGylated PEI-cholesterol lipopolymers (Figure 5b) were synthesized, where cholesterol functionalization was helpful for endocytosis, enhancement of drug loading capacity and reduction in the drug burst release [40]. When they were used as nanocarriers, SF-loaded NPs exhibited maximum drug loading (13.1% ± 2.65) and concentration-dependent toxicity by pharmacological action through the charge shielding mechanism.

An amphiphilic brush copolymer PHEA-BIB-ButMA (PBB, Figure 5c) was synthesized by Atom Transfer Radical Polymerization (ATRP) [41]. SF was entrapped into its inner hydrophobic core in an aqueous environment as a drug reservoir. The spherical morphology (200 nm size), negative ζ potential and sustained SF release capability was found. Compared to free drug, SF-loaded NPs showed a higher inhibition efficacy of tumor growth and more accumulation in the solid tumor mass in in vivo xenograft models.

Liquid crystalline nanoparticles (LCN) have high stability, sustained drug release, and high drug payload to the target site [42]. Thapa et al. prepared a pH-responsive polymer-coated LCN with poly-L-lysine (PLL) and poly(ethylene glycol)-*b*-poly(aspartic acid) (PEG-*b*-PAsp) for SF delivery [11]. Due to selective depolymerization of polymer layers by acidic tumor microenvironment, the pH-responsive SF-loaded nanomedicine showed enhanced drug release. As a result, the intracellular uptake of SF was significantly improved, generating better antitumor efficacy against HepG2 cells.

The amphiphilic polymer based on polyacryic acid (PAA) with D-α-tocopherol succinate (VES) via a disulfide bond linker was synthesized [43]. SF was entrapped in the polymer micelles, where the release of SF was dependent on the concentration of glutathione (GSH) because of GSH sensitivity of disulfide linkage. A 2.8-fold bioavailability of SF was achieved by injecting the animal compared with free SF by this redox-responsive SF delivery system.

To control particle morphology, size distribution and reproducibility from batch-to-batch, a microfluidic process with glass-based microcapillary devices was developed for preparation of micellar-like PEG-*b*-PCL NPs loaded with SF free base [44]. Under optimized nanoprecipitation process, spherical NPs with loading degree (16%), encapsulation efficiency (54%), particle size less than 80 nm were obtained. More importantly, 20,000-fold increase in solubility was achieved as compared to free SF in PBS. They found process temperature, flow rate, nanoprecipitation temperature, and organic solvent removal method had the significant effects on particle size distribution, morphology distribution, zeta-potential, drug loading, encapsulation efficiency and process yield. The in vitro drug release study indicated that SF was released from the NPs in a sustained manner, where 13.6% and 25.3% release rate in 8 and 24 h at pH 7.4 was shown.

**Figure 6:**
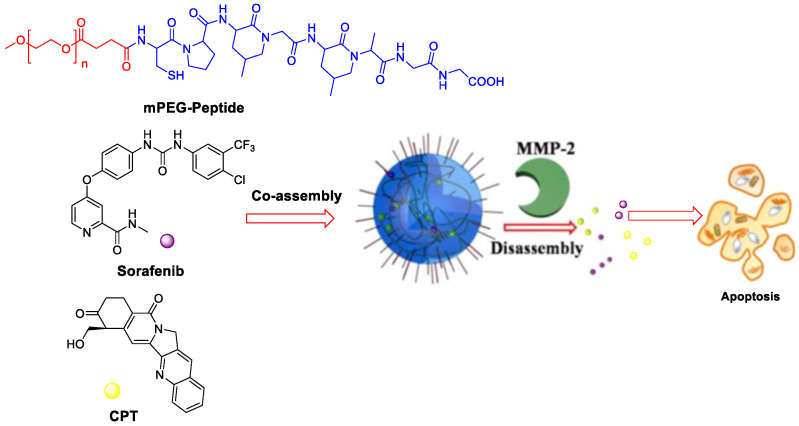
MMP-2-responsive nanoparticles for synergistic antitumor effect of anti-angiogenesis and chemotherapy. Adapted with permission from [45]. Copyright 2016, American Chemical Society.

Colorectal cancer has overexpressed matrix metalloproteinase (MMPs), especially MMP-2. The MMP-2 responsive nanocarrier assembled by amphiphilic polyethylene glycol (PEG)-peptide copolymer for simultaneously loading camptothecin and SF for combination therapy (Figure 6) [45]. Two drugs were released upon exposure to tumor microenvironment because the peptide segments could be digested by MMP-2, leading to higher synergistic efficacy than that of single antitumor agents. The photoacoustic (PA) imaging signals kept constant for the whole week during detection the angiogenesis of in vivo HT-29 bearing mice.

### 2.3. Inorganic and Metal Nanocarriers

Hollow mesoporous silica nanoparticles (MSNs) show uniform particle size distribution, excellent water dispersion, large specific surface area and good biocompatibility [46], which are ideal drug delivery nanocarriers [47]. Shao’s group reported a series of MSNs-based SF NPs [48,49]. For example, MSNs were used as nanocarriers for the co-delivery of SF and siRNA, leading to enhanced cytotoxicity and improved the tumor target of SF in asialoglycoprotein receptor (ASGPR)-overexpressing Huh7 cells [48]. Since lactobionic acid (LA) could target human HCC effectively, an ASGPR-targeting SF delivery system based on MSNs co-delivery of vascular endothelial growth factor (VEGF)-targeted siRNA (siVEGF) and LA was synthesized as follows [48]. NPs based on MSN-NH_2_ and SF were fabricated by non-covalent interactions. Then, LA was chemically linked to the surface of the NPs. Finally, siVEGF were loaded into the nanocarrier by electrostatic interaction. The improvement in the targeting property resulted in enhanced anti-cancer efficacy of SF. Recently, SF was loaded into manganese-doped MSNs by physical adsorption [50]. In this case, high-concentration GSH could break manganese-oxidation bonds leading to the degradation of MSNs. This dual GSH-exhausting (consumption or synthesis inhibition of GSH) nanomedicine showed a significantly enhanced in vitro inhibitory effect.

To decrease the risk of post-surgical HCC relapse, a wound-targeted nanodrug containing immune checkpoint inhibitor (anti-PD-L1) and SF as an angiogenesis inhibitor was developed, where a mesoporous silica with a surface-coated platelet membrane as surgical site-targeting nanocarriers was used [51]. The resulting nano-formulation efficiently inhibited post-surgical HCC relapse without obvious side effects.

Gold NPs with high biocompatibility and negligible toxicity, small size and precise targeting ability are suitable candidates for a drug delivery system for the treatment of various diseases. FA-*b*-PEG-modified gold NPs loaded with SF tosylate were fabricated, providing an effective treatment for retinal neovascularization in patients of diabetic retinopathy [52]. MiR-221 plays a role in promoting tumorigenesis in HCC by inhibiting the expression of p27. Cai et al. investigated the synergistic anti-tumor effects of SF and gold NPs-loaded anti-miR221, leading to a strong synergistic effect on inhibiting proliferation of HCC cells [53]. Recently, alginate/CaCO_3_ hybrid loaded with SF tosylate and gold hexagons was developed for the dual (chemo-radio) treatment of HepG2 cells [54].

As a versatile inorganic compound, sodium selenite (Na_2_Se) NPs exert anticancer effect by inducing apoptosis. A co-delivery system for SF and Na_2_Se was prepared when tripolyphosphate was used as a crosslinker via the solvent evaporation technique [55]. The resulting NPs had a zeta potential of −37.5 mV and size (diameter) in the range of 208 nm to 0.2 µm. The sustained release of the drug to elicit synergetic action was developed. Selenium NPs have distinct characteristics of high biocompatibility, low-toxicity and degradability in vivo, and radiosensitization effects with X-ray. An injectable thermosensitive nanosystem based on SF, Se NPs and PLGA-PEG-PLGA was prepared [56]. When it was injected in HepG2 tumor-bearing nude mice, the resulting hydrogel for long-acting therapy with the degradation of PLGA-PEG-PLGA was obtained. The raised expression of cleaved caspase-3 in tumor tissue was observed based on the synergistic local treatment and chemoradiotherapy.

ZnO-NPs possess excellent physicochemical properties of safety, biodegradability, fast delivery rate and cytotoxicity against tumor cells [57]. ZnO-NPs might be a safe candidate in combination with SF to generate enhanced treatment effect [58].

### 2.4. Co-Delivery of SF and Other Drugs

**Figure 7:**
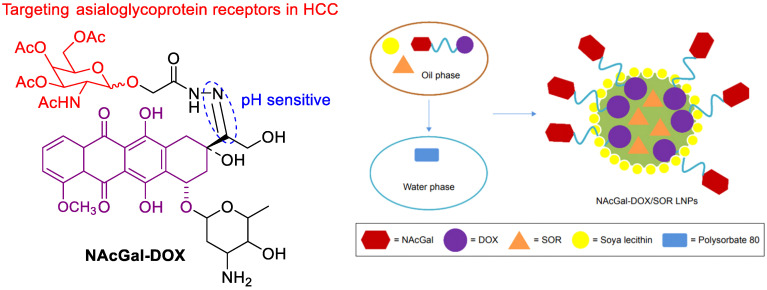
The chemical structure of NAcGal-DOX and co-loading in NAcGal-DOX/SF NPs.

The SF-based monotherapy is often hampered by its modest efficacy, serve systemic toxicity and high occurrence of drug resistance. In order to enhance the antitumor effect of SF and reduce its side effects, different drugs are co-loaded in nanomedicine. Doxorubicin (DOX) is an effective chemotherapy agent, but it suffers from some drawbacks such as low response rate, severe side effects and cytotoxicity to normal tissues. Although DOX and SF have completely different chemical characteristics, Babos et al. reported the entrapment of DOX and SF by PLGA/PEG-PLGA using the double emulsion solvent evaporation method [59]. The DOX-SF NPs showed a suitable size (~142 and 177 nm), high drug encapsulation efficiency (69% for DOX and 88% for SF) and high drug loading. The DOX was released continuously within 6 days, while the SF was released quickly during 24 h under biorelevant conditions. Duan et al. formulated a specific sugar-modified pH sensitive lipid system for the co-delivery of DOX and SF for combination HCC chemotherapy [60]. In this case, DOX prodrug (NAcGal-DOX, Figure 7) contained pH-sensitive hydrazine moiety and N-acetylgalactosamine, allowing a rapid DOX release at the tumor site and targeting to highly expressed asialoglycoprotein receptors in HCC. DOX and SF were entrapped by single-step nanoprecipitation in the presence of soya lecithin and polysorbate 80. The targeted ability of sugar to HCC and strong synergy between DOX and SF generated a higher anti-tumor efficiency of drugs.

In order to deliver SF in a targeted fashion into undifferentiated/anaplastic thyroid carcinoma cells, Mato et al. developed SF-loaded PLGA NPs covalently attached with cetuximab [61]. SF-loaded PLGA NPs was firstly prepared by the single emulsion evaporation method. Then, EDC/Sulfo-NHS cross linking chemistry was utilized to conjugated cetuximab to SF-loaded PLGA NPs, yielding 51% cetuximab incorporation. The resulting NPs showed suitable size (252 nm) and drug entrapment efficiency (58%). A higher specificity to the anaplastic cells line (CAL-62) that overexpress EGFR was shown, providing a promising targeting approach for the treatment of epithelial thyroid cancer.

Recently, SF and all-trans retinoic acid (ATRA), a differentiation-promoting drug, were loaded into PEG–PLGA polymer micelles [62]. The two drugs-loaded micelles exhibited relatively slow drug release and effective cell uptake, yielding significant antitumor effect and minimal systemic toxicity toward the FTC-133 thyroid cancer-bearing BALB/c nude mouse model.

Combretastatin-A4 (CA4) is a representative vascular disrupting agent (VDA) which causes significant central tumor necrosis by selectively arresting tumor blood flow and disrupting the established tumor vasculature. Wang and coworkers prepared NPs of poly(L-glutamic acid)-graft-methoxy poly(ethylene glycol)/combretastatin A4 sodium salt (CA4-NPs) via a Yamaguchi reaction and following salination reaction [63]. The combination of SF 30 mg.kg^−1^ + CA4-NPs 30 mg.kg^−1^ (on the CA4 basis) showed an over 90% tumor suppression rate in a hepatic H22 subcutaneous tumor model with low systemic toxicity. A total of 71% of treated mice survived in tumor-free state for 96 days. These findings indicated that the two-pronged attack of SF and CA4-NPs (as a tumor blood-flow reducer) was a promising therapeutic approach for HCC treatment.

The combination of SF and Bosutinib drug was co-loaded in a self-emulsified drug delivery system [64]. The superior antitumor treatment effect for HCT116/SW1417 by destroying the DNA structure to inhibit cell proliferation was shown. Yin et al. formulated liposomes entrapping ceramides and sorafenib and showed a synergistic cytotoxic effect on HepG2 when compared to single-drug liposomes [65].

Most nanoformations focus on one nanocarrier for one drug. Sukkar et al. investigated a comparative study on two polymeric nanocarriers for SF against HepG2 Cells [66]. In this study, two biocompatible polymers (PLGA or PCL) were used for the co-delivery of SF tosylate and gold nanoparticles (G), where the latter was selected as a radiosensitizer. SF/PCL NPs showed slower SF release rate than SF/PLGA NPs (27.9% vs. 31.2% in pH = 7.2 and 46.3% vs. 61.3% in pH = 5.2). Both SF/PCL/G NPs and SF/PLGA/G NPs exhibited elevated necrosis and apoptotic levels through the synergistic combined chemo–radio treatment. The IC_50_ vales for SF/PCL NPs, SF/PLGA NPs, SF/PCL/G NPs, and SF/PLGA/G NPs were found to be 12.7, 10.3, 8.9, and 4.9 µg/mL, respectively.

### 2.5. Imaging-Guided SF Nanodrugs

Nanomedicines combining drug delivery and imaging functions have been employed as theranostic tools for imaging-guided cancer therapy. Superparamagnetic iron oxide nanoparticles (SPIONs) are suitable nanocarrier systems for cancer therapeutics [67,68,69]. SPIONs within the lipid matrix were used to construct magnetically responsive SLNs. Grillone et al. developed SF-loaded magnetic SLNs (SF-Mag-SLNs) to enhance SF delivery with the help of a remote magnetic field using cetyl palmitateas lipid matrix [70]. The resulting NPs had a 90% SF loading efficiency, a regular spherical shape and an average diameter of less than 300 nm. SF-Mag-SLNs were able to inhibit cancer cell proliferation through the SF cytotoxic action. Good targeting properties and attenuated side effects were achieved via a magnetically driven accumulation of SF. However, the preparation process is extremely complex and tedious.

The use of poly(vinyl alcohol)-coated SPIONs as a polymeric-magnetic delivery system for SF with significantly enhanced activity was developed via co-precipitation and physical entrapment methods [71]. An entrapment value of 76.37% and a release rate of SF up to 66.7% in 80 h were obtained. The MTT assay showed that the cytotoxicity of SF-loaded PVA/SPIONs was comparable or higher than that of free SF. The apoptosis study in HepG2 cells indicated that the combination of SF with PVA/SPIONs showed a higher percentage of early apoptotic cells than free SF (13.8% vs. 11.4%).

A reduction and pH dual-sensitive nano-formulation co-encapsulating SPIONs and SF was developed and further functionalized by anti-GPC3 antibody (AbGPC3) for magnetic resonance imaging (MRI)-monitorable HCC-targeted therapy [72]. The AbGPC3-mediated active targeting enhanced cancer cell uptake and tumor accumulation of nanodrug. Consequently, the nanodrug displayed prominent anticancer effects (the lowest tumor weight was only 12.3% of that for the saline group) in an animal study via responding to the cytoplasmic glutathione and lysosomal acidity. Moreover, tumor detection and monitoring of drug delivery process by MRI were obtained.

**Figure 8:**
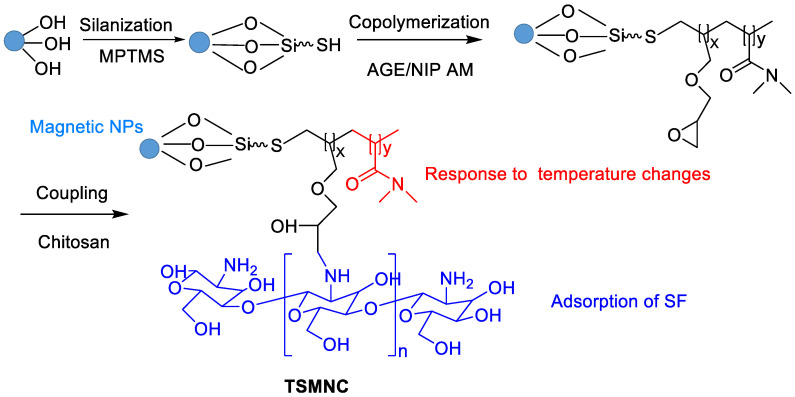
Synthetic route of TSMNC.

Faramarzi et al. developed functionalized magnetic nanoparticles with chitosan for adsorption of SF and thermosensitive N-isopropylacrylamide to control release (Figure 8) [73]. The radical copolymerization of allyl glycidyl ether (AGE), N-isopropylacrylamide (NIP) and silica-coated magnetic NPs was firstly involved. The following coupling reaction between chitosan and epoxy ring of the AGE afforded thermosensitive magnetic nanocarrier (TSMNC). Due to superparamagnetic properties, rapid adsorption and slow release at low and high temperatures, Fickian diffusion-controlled drug release was obtained, where about 88% of SF was released within 35 h at 45 °C. Recently, SF tosylate-loaded PCL-superparamagnetic nanoparticles were prepared by an emulsion solvent evaporation method and optimized using Box–Behnken design [74].

Zhang et al. prepared SF and gadolinium (Gd) co-loaded liposomes (SF/Gd-liposomes) using Lipoid E80 and cholesterol as carriers for MRI-guided visualization of the delivery and HCC treatment [75]. The solubility of SF in SF/Gd-liposomes was significantly increased to 250 ng/mL. SF/Gd-liposomes exhibited spherical shapes or ellipsoidal shapes, uniform particle size distribution, negative zeta potential, high encapsulation efficiency and drug loading. Due to its limited drug release and slower absorption by HepG2 cells, lower cell cytotoxicity from SF/Gd-liposomes was observed. In the MRI test, longer imaging time and higher signal enhancement were obtained at the tumor tissue.

Manganese dioxide (MnO_2_) nanosystems can react with H^+^ and glutathione (GSH) in the tumor microenvironment (TME) [76]. The resulting paramagnetic Mn^2+^ significantly increases the contrast of T_1_ in MRI, which is useful for tumor-specific imaging and multifunctional drug carrier systems [77]. Wang et al. reported aptamer-mediated hollow MnO_2_ for targeting the delivery of SF, where aptamer could specifically bound to glypican-3 (GPC3) receptors on the surface of HCC [78].

A dicarboxylic acid-containing ICG was selected as a NIR photosensitizer and fluorescent dye, which was co-loaded with SF by Pluronic F127 to produce self-assembled and self-monitored nanodrug [79]. Through a strong hydrogen bond between the carboxyl group of ICG and the urea bond of SF, stable nanodrug with a particle size of ~70 nm was formed. Due to optimal physical and chemical characteristics, nanodrugs had a longer blood retention time (up to 36 h) and preferable tumor accumulation, thereby yielding ultrasensitive NIR-II fluorescence images and photothermal tumor ablation (ΔT = 25 °C) in in vivo nude mice bearing Huh7 tumor. The combination of photothermal therapy and chemotherapy based on ICG and SF would offer potential for HCC treatment since two drugs and Pluronic F127 have been widely used in the clinic.

Liu et al. designed a new nanocarrier platform for SF delivery, where the tumor chemotherapy process was guided and evaluated by NIR-II dual-modal optical coherence tomography and photoacoustic imaging [80]. Because integrin α_γ_β_3_ is regarded as the targeted site for HCC, arginine–glycine–aspartic acid (RGD) functionalized carriers are promising. The multifunctional SF theranostic nanosystems were prepared based on cyclic RGDfK and S-Cy5.5 fluorescent dyes-functionated PEG-PCL for fluorescence imaging-guided HCC treatment [81].

### 2.6. Actively Targeted Nanosystems

**Figure 9:**
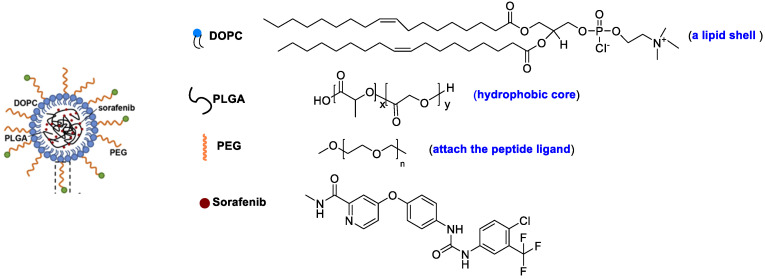
A lipid-coated nanocarrier.

Feng et al. designed a lipid-coated nanocarrier functionalized with peptides specific for glypican-3, which showed targeted drug delivery to human HCC xenograft tumors [82]. PLGA and 1,2-dioleoyl-snglycero-3-phosphocholine (DOPC) were utilized as a hydrophobic core to entrap SF and as a lipid shell to prevent drug leakage, respectively (Figure 9). The peptides are attached to the nanocarrier surface via a thioether-mediated conjugation. The entrapment efficiency (%EE) for SF was > 80% with concentrations up to 69.5 mg/L, which showed a 1900-fold improvement in aqueous solubility versus free SF. The half-life release ratio in vitro was 22.7 h. The greater tumor regression was shown due to the target peptide versus controls after 21 days of therapy.

**Figure 10:**
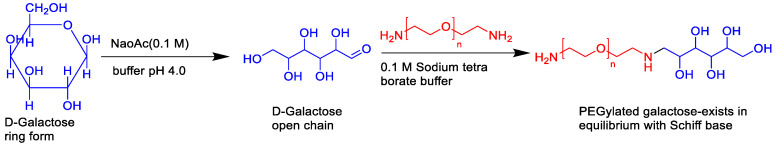
The synthetic route of PEGylated galactose.

SLN appended with PEGylated galactose (Figure 10) was developed to modulate the site-specific oral delivery of SF for HCC treatment because galactose is a C-type lectin receptor binding ligand [83]. The encapsulation of SF into SLN and use of PEGylated galactose as targeting ligand provided a liver-targeting, biocompatible drug delivery system and increased circulation half-life. The oral bioavailability of the SF can be improved by encapsulating it in SLN and grafting it with PEGylated galactose.

Zhong et al. reported a redox-responsive SF delivery system based on apolipoprotein E peptide-decorated disulfide-cross-linked polymer and SF by self-assembly and self-crosslinked process [84]. The resulting NPs exhibited a good SF loading of 7.0 wt%, a small size (37 nm) and high stability. Due to presence of a disulfide bond, reduction-triggered drug release from polymer nanocarrier, a long circulation time and enhanced SF therapy for orthotopic HCC in mice were shown. AMD3100 possess anti-tumor activity upon combination with chemotherapy. The resistance to anti-angiogentic therapy of HCC by SF is activated by C-X-C receptor type 4 (CXCR4). Chen et al. co-delivered SF together AMD3100 attached to lipid-coated PLGA NPs [85]. They demonstrated that CXCR4-targeted NPs efficiently delivered SF into HCCs and human umbilical vein endothelial cells (HUVECs) to achieve cytotoxicity and anti-angiogenic effect in vitro and in vivo.

**Figure 11:**
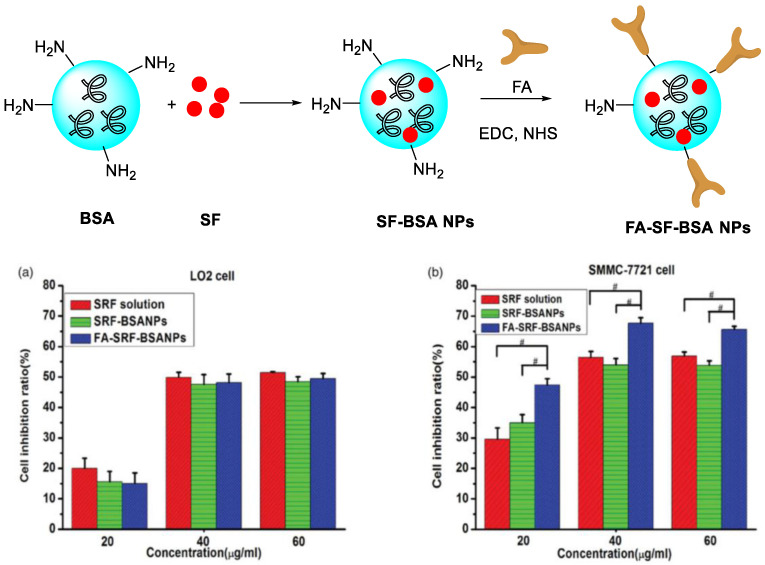
Schematic synthetic route of SF/FA/PEG-PLGA NPs. Cell inhibition ratio on three concentration levels of SF solution, SF-BSA NPs, and FA-SF-BSANPs against (**a**) LO2 cell lines or (**b**) SMMC-7721 cell lines after incubation for 24 h [88]. ^#^ indicates a statistically significant difference between two groups *p* < 0.05, independent sample *t*-test. Copyright 2021, Taylor & Francis.

Folate (FA) can enter cells through a receptor-mediated endocytosis pathway. It is highly expressed in tumor tissue at a level 100–300 times higher than that in normal tissues. The FA receptor is a good target for targeting delivery systems for many solid tumors. Zhang et al. prepared FA-functionalized polymeric micelles loaded with SPIONs and SF, which can increase the concentration of SF in HepG2 cells [86]. Li et al. developed SF/FA/PEG-PLGA NPs with both anticancer and magnetic resonance properties, which can improve the antitumor activity in vitro [87]. In 2019, Xie et al. developed bovine serum albumin (BSA) modified with FA by chemical coupling as the drug carrier (Figure 11) [88]. Good stability for more than 1 month at room temperature was found. The average particle size (158 nm), zeta potential (−16.27 mV), entrapment efficiency (77.25%), and drug loading (7.73%) were obtained. As compared with SF-BSA NPs and SF solution, SF-BSA-FA NPs showed the best intracellular uptake of hepatoma cells (SMMC-7721) and the strongest inhibitory effect. Moreover, excellent tumor targeting ability with higher C_tumor_/C_blood_ value (0.66) than those of the former two (0.56 and 0.41) in a nude mice liver cancer model were found.

FA-modified PEG-nitroimidazole grafts exhibited a targeting SF delivery ability in the treatment of hypoxic HCC [89]. Since antibody hGC33 has important antitumor activity, Shen et al. described hGC33-modified PEG-b-PLGA for SF delivery to increase the targeting to HCC cells [90].

The presentative SF-based drug delivery systems are summarized in Table 1.

### 2.7. Combination of Ferroptosis and Others

Ferroptosis is an alternative to the traditional apoptotic pathways for cancer therapy, which is regarded as an iron-based Fenton-type reaction. Since SF can induce the occurrence of ferroptosis, some combinational therapy methods have been proposed [91]. Zhou et al. described a metal-polyphenol network-based SF nanodrug for combinational ferroptosis and PDT, showing a more excellent inhibitory effect on tumor cells [92].

**Figure 12:**
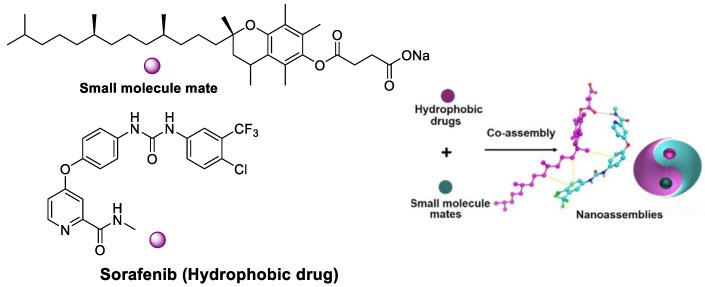
SF/D-α-tocopherol succinate nanoassemblies based on the drug–mate strategy.

The novel drug–mate strategy has been developed in the nanomedicine field. Namely, amphiphilic small molecule and hydrophobic drugs with poor solubility are selected as a mate and drug, respectively (Figure 12). Taking SF/D-α-tocopherol succinate nanoassemblies as an example, drug loading content (46%) was shown compared with the lipid-based SF nanodrugs (11%) and SF liposomes (4%) [93]. The highest SF water dispersion was due to stronger interaction between small molecular mate (SMA) and SF than that between SF itself. In vitro cytotoxicity assay indicated the IC_50_ of SF NPs based on the drug–mate strategy was close to that of free SF (3.44 vs. 4.44 μg/mL). However, the pharmacokinetics of the former were largely improved, where the plasma concentrations of SF was 2 and 20 times than that of the SF solution and SF suspension, respectively. In vivo antitumor efficacy study further revealed the increased bioavailability and enhanced therapeutic efficacy of SF.

## 3. Challenges and Opportunities

With the development of nanotechnology, nanocarrier-based SF delivery systems have been widely studied. Its poor pharmacokinetic properties have been improved to some extent. The synergistic drug combinations also exhibit potential for cancer treatment due to their superior therapeutic benefits to the classical monotherapeutics. The innovative optimization of nanocarriers structures (modulation of particle size, an increase in particle-specific surface area, multifunctional surface modification, etc.) improves the bioavailability and therapeutic efficiency of anti-tumor drugs. The chemical and physiological properties of nanocarriers largely affect the synergistic actions combined drugs. However, some challenges still exist such as low drug loading, uncontrollable drug release, insufficient tissue penetration, loss of targeting ability, biosafety, etc. In particular, ligand- or surface-modified nanocarriers are utilized to enhance the SF anti-tumor effect. According to the receptors over-expressed on the surface of tumor cells, specific ligands, such as antibodies, proteins, peptides, aptamers or folic acid, were introduced for the surface modification of nanocarriers to achieve active targeting via biological substrate/receptor (antibody/antigen) recognition. However, the “overexpression” of relevant receptors on the surface of tumors is not sufficient to impart high selectivity due to tumor heterogeneity. Moreover, the development of nanoformulation often requires complex nanocarrier synthesis/modification, purification and fabrication. Some shortcomings such as stability, handling inconvenience, blood stability, reproducibility from batch-to-batch and scale-up issues are encountered. The reproducibility from batch to batch is also a problem. Most SF-based nanodrugs are studied at the cellular and animal level. There are various physiological barriers in terms of clinical translation.

## 4. Conclusions

The selection of nanocarriers for SF delivery is essential to improve its biopharmaceutical properties. Various types of representative materials such as lipid- and polymer-coated nanomatrix, natural and synthetic polymeric, MSNs, pH- or GSH- responsive NPs, gold NPs, injectable sodium selenite NPs and in situ gel with lipid coating are utilized to promoting SF nanoformulations. The bioavailability, solubility, drug targeting and cytotoxicity of SF are improved, which is facile for circumventing some of the unfavorable outcomes of traditional SF regimens. SF-based nanodrugs cannot stay in the tumor site for a longer period when they enter the blood circulation by diffusion and via the ERP effect. Moreover, some nanocarriers are difficult to be removed from the blood stream due to high physicochemical stability and unsuitable particle size, which may increase systemic toxicity and organ damage. Appropriate surface modifications could enhance the localization and specific accumulation of nanocarriers in tumor cells by ligand–receptor interactions. Studies have shown that precise targeting of receptors on tumor cells or tumor vessels by ligand-modified nanocarriers can improve the efficacy of drugs. The application of biodegradable nanocarriers would be a promising strategy to control toxicity and improve biological compatibility. In the future, more attention should be paid to the surface characteristics, biosafety, targetability by connecting tumor-targeting ligands, controlled release with stimuli-responsive linkers, and so on. Moreover, it is important to combine SF with other treatments for synergistic therapy (chemotherapy, immunotherapy, photodynamic therapy, photothermal therapy). Scientists should make more efforts to investigate the biodistribution, pharmacokinetic response and in vivo efficacy of various SF nanoformulations. All in all, SF nanoformulations hold great potential and prospects in cancer therapeutics in the future. It is urgent to study the mechanisms underlying how SF interacts with cellular molecules and other drugs to increase its efficacy and reduce resistance in HCC patients.

## Figures and Tables

**Table 1:** The presentative SF-based drug delivery systems.

NPs	Average Particle Size (nm)	Zeta Potential (mV)	Entrapment Efficiency (EE%)	Drug Loading (DL%)	Application	Ref
Cyclodextrin @SF	200	−11	100	17	HCC cell line	[35]
PEI-cholesterol @SF	30	12.4	-	13.1	HepG2 cells	[40]
PLGA@ cetuximab @SF	277	−11.1	-	-	CAL-62 and Nthyori 3-1 cell	[61]
PEG-PLGA @SF	230	-	-	15%	IC_50_: 2.6 μM (HSC); IC_50_: 1.6 μM (HUVEC); liver fibrosis of C3H mice	[39]
Glypican-3@SF	114	−20.9	>80%	[SF]: 69.5 mg/L, T_1/2_ = 22.7 h	HCC cells in vitro, growth inhibition of HCC tumors	[82]
SPIONs @SF	5–15	-	76.37%	-	HCC cells, Wistar rats	[71]
PEGylated galactose @SF	111	−19.8	95	-	HepG2 cells and BALB/c mice	[83]
lipid@SF	221	−37	100	18.46	Four different HCC cell lines	[22]

## Data Availability

Not applicable.
